# DNA repair gene expression is associated with differential prognosis between HPV16 and HPV18 positive cervical cancer patients following radiation therapy

**DOI:** 10.1038/s41598-020-59383-8

**Published:** 2020-02-17

**Authors:** Klarke M. Sample

**Affiliations:** The National Health Commission’s Key Laboratory of Immunological Pulmonary Disease, Guizhou Provincial People’s Hospital, The Affiliated Hospital of Guizhou University, Guizhou, China

**Keywords:** Double-strand DNA breaks, Cervical cancer

## Abstract

Cervical cancers are almost always induced by HPV infections, of which HPV16 and HPV18 are predominant. Cancers associated with these strains are induced through DNA repair factors and have a differential response to radiation therapy. Hence this study focuses on finding DNA repair gene expression differences in HPV16 and HPV18 positive cervical cancers after radiation therapy. A higher number of somatic mutations were observed in HPV16 positive cervical tumours for patients that were disease free when compared to those who recurred/progressed. Moreover, hierarchal clustering of RNAseq data from The Cancer Genome Atlas was conducted to identify groups of DNA repair genes associated with a differential prognosis for cervical cancer following postoperative radiation therapy. TP53BP1, MCM9 (at higher than mean levels), POLR2F and SIRT6 (at lower than mean levels), were associated with an increase in patients experiencing cervical cancer recurrence/progression following postoperative radiation therapy when HPV18 positive, but not HPV16 positive. The expression patterns of these genes provide an explanation for the higher rate of postoperative radiation therapy resistance associated with HPV18 positive cervical cancer patients. Therefore, HPV18 positive cervical tumours may be more likely retain a greater non-homologous end joining and homologous recombination pathway activity, which could dampen the effect of postoperative radiation therapy. Moreover, greater susceptibility to postoperative radiation therapy could be caused by the reliance of cervical cancer cells upon the single-strand annealing and nucleotide excision pathways for repair of DNA damage.

## Introduction

Worldwide, cervical cancer ranks forth for incidence and mortality for women, with 3.2% of new cancer cases and 3.3% of total cancer deaths^1^. It has been estimated that 99.7% of all cervical cancers are associated with Human papillomavirus {HPV} infection^2^. Several hundred strains of HPV that infect mucosal and cutaneous epithelia are known to exist (although not all have fully characterized genomes), which share some genetic features, such as a circular double-stranded DNA genome^3^. Of these strains, HPV16 and HPV18 are associated with a particularly high cancer risk and are the most commonly associated with cervical cancer (detected in approximately 70% of cervical cancers), though HPV16 is generally thought to be the most prevalent (HPV16 detected in 50% of cervical cancers and HPV18 in 20%)^3–5^.

Currently, effective HPV vaccines are available^4,6^, which have contributed to a decline in cervical cancer rates in developed counties^1^. In theory these vaccines could eliminate the majority of cervical cancers. However, there are still individuals who are (and will likely continue to) be infected with HPV for a myriad of reasons, e.g. because they were infected before vaccine availability or are unable to (or choose not to) receive the vaccine. Hence, cervical cancer rates have remained relatively high in many Sub-Saharan African counties, where it is the currently the most diagnosed and leading cause of cancer death^1^. Therefore further research into the etiology of HPV associated cervical cancer and the development of improved treatment regimens is important.

A commonly used therapeutic strategy for cervical cancer involves the administration of radiation therapy following the surgical removal of tumours^3^. Indeed, HPV associated tumours have been linked to a relatively high chance of responding favourably to radiation therapy^7^ and previous research has demonstrated that the HPV16 E2 protein can influence the radiosensitivity of cervical keratinocytes^8^. Nevertheless, the favourable response to radiation therapy has not been observed to be universal and some subgroups have a poorer outcome^9^. Compared to HPV16+ cervical cancer, HPV18+ tumours have been shown to be associated with more a more aggressive phenotype and a poorer outcome following radiation therapy^10–14^.

The viral oncogenic proteins encoded by HPV16 and HPV18 are known to target and degrade DNA repair factors such as the retinoblastoma protein family and TP53, which contributes cervical cancer development^3,4^. Due to the aforementioned link between HPV associated carcinogenesis and the aforementioned DNA repair proteins, the hypothesis explored in the study is that ‘differences in DNA repair gene expression patterns between HPV16 positive {HVP16+} and HPV18 positive {HPV18+} cervical cancers are connected to the poorer prognosis for HPV18+ patients following postoperative radiation therapy {PRT}’. RNA sequencing {RNAseq} data from The Cancer Genome Atlas {TCGA} Cervical Cancer Study^15^ was utilized to explore the hypothesis. The Cervical Cancer Study contained RNAseq data for 202 surgically resected cervical cancers and clinical data including whether PRT was conducted and whether the cervical cancer had recurred/progressed.

## Methods

### DNA repair database

A database of 569 DNA repair genes was constructed using gene lists from the Gene Ontology (GO) project: DNA Repair (GO:0006281); Homology directed repair (GO:0000724); Non-homologous end joining (GO:0006303); DNA Damage Checkpoint (GO:0000077); Mitotic G2 DNA damage checkpoint (GO:0007095); Mitotic G1 DNA damage checkpoint (GO:0031571); intra-S DNA damage checkpoint (GO:0031573). The derivative gene list was filtered to retain the genes that were annotated as being present in *Homo sapiens*, the gene list was also curated to ensure the official gene symbols and Entrez Gene-IDs were up-to-date. Furthermore, all duplicate entries were identified and removed using Unix uniq, and annotated with the UCSC Table Browser and NCBI Batch Entrez.

The aforementioned gene list was subsequently used to download TCGA RNAseq data for patients in the Cervical Squamous Cell Carcinoma and Endocervical Adenocarcinoma project (provisional dataset) using cBioPortal^16,17^. RNAseq z-Score data (where the gene expression for each patient is described as a standard deviation {SD} from the mean expression of the cohort for each gene) was available for 546 of the 569 DNA repair genes. The expression data for each patient was then paired with data from cBioportal regarding their genomic integrity (somatic mutation number and fraction of genome altered), postoperative radiation therapy status (TCGA does not report the form of postoperative radiation therapy, merely whether the patients either received the therapy or not) and disease status (disease free {DF} or recurred/progressed {RP}). Additionally, the HPV16/HPV18 status for each patient was added using data from supplementary files attached to the following articles: The Cancer Genome Atlas Research Network^15^ (HPV status called using genomic sequences, e.g. exome sequencing data) and Banister *et al*.^18^ (HPV status called using transcribed sequences, i.e. RNAseq data). Patients with strains other than HPV16 or HPV18 were not analyzed due to their low representation within the cohort. Moreover, patients were excluded if the HPV calls were in disagreement between the two sources or if multiple HPV strains were called within one patient. The final database (Supplementary Table 1) included data for a total of 202 patients, of which 164 were HPV16+ and 38 were HPV18+ (Supplementary Fig. 1).

### Data analysis

Cluster 3.0^19^ was used to perform hierarchal clustering of the RNAseq z-Score data using a Spearman rank correlation with complete linkage. The clustered data was subsequently graphed in the form of a heatmap using Java TreeView^20^. All other statistical analyses were performed and graphed using GraphPad Prism (version 8), the specific statistical test performed (Fisher’s Exact Test or Mann-Whitney U test) is detailed within each respective figure legend. The significance of the data is described within each figure using the following standardized annotation: ns P = not significant, *P = <0.05, **P = <0.01, ***P = <0.001, ****P = <0.0001. All statistical tests were two tailed, unless stated otherwise.

### Ethical approval and consent to participate

All patient data used in this study is publically available, required no additional informed consent for participation and was conducted in compliance with TCGA guidelines. It is noteworthy that participation in the Cervical Cancer Study (TCGA) required informed consent, which was collected by the NCI/NHGRI. Additional ethical approval was not required from the Guizhou Provincial People’s Hospital for this study. In totality, this study was performed in accordance with the Declaration of Helsinki.

### Consent for publication

The author declares that this work has not been published (in full or in part), nor is it under consideration for publication elsewhere. The work contained within was conducted and approved for submission by the author.

## Results

**Figure 1:**
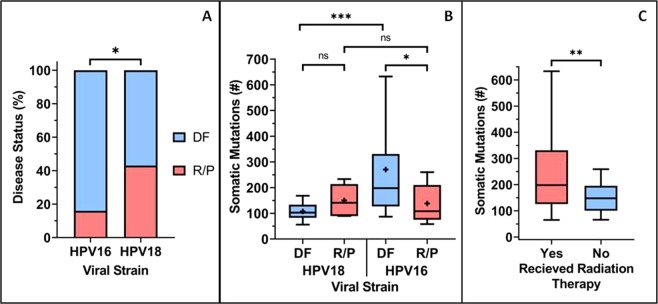
Increased survival of HPV16+ patients correlates to increased somatic mutation levels. (**A**) TCGA patients who received PRT were significantly (using Fisher’s exact test) more likely to experience a DF status if HPV16+ compared to HPV18+ patients. (**B**) Significantly (using the Mann-Whitney U test) higher levels of somatic mutation were observed in patients who were HPV16+ and DF after receiving PRT when compared to both HPV16+ patients who later RP and HPV18+ patients who were DF. There was no significant (ns) difference in somatic mutation level between HPV18+ patients who were DF when compared to those who RP. (**C**) DF HPV16+ patients who received PRT had significantly (using Mann-Whitney U test) higher somatic mutation rates than those who did not receive PRT.

In accordance with the scientific literature discussed earlier, patients who received postoperative radiation therapy in the Cervical Cancer Study cohort were more likely to experience disease recurrence/progression if HPV18+, when compared to HPV16+ patients (Fig. 1A). Moreover, the number of somatic mutations was observed to be significantly higher in HPV16+ cervical cancer patients who were DF after receiving PRT when compared to HPV18+ patients (Fig. 1B) and HPV16+ patients who were DF despite not receiving PRT (Fig. 1C). Whilst there was a slightly higher rate of copy number variation (as measured by the fraction of genome altered) in the HPV18+ tumours relative to the HPV16+ tumours (Supplementary Fig. 2A), there was no significant difference in the copy number variation between HPV16+ and HPV18+ patients in the context of PRT (Supplementary Fig. 2B).

**Figure 2:**
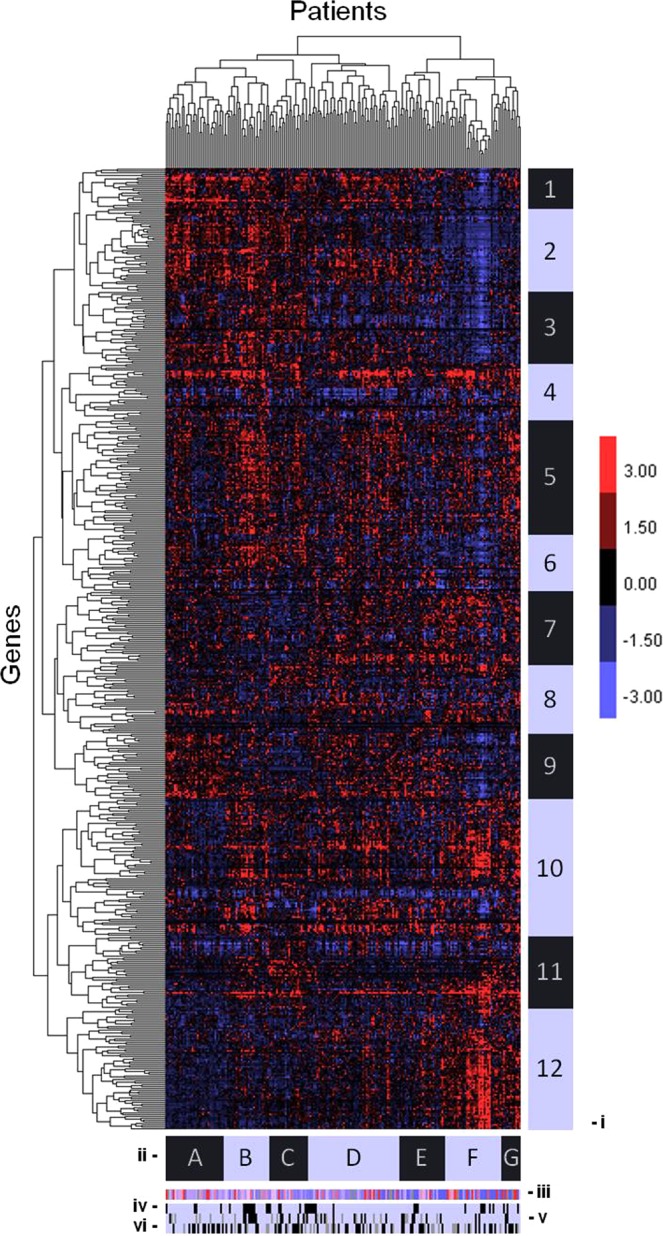
Clustering of DNA repair genes expression in cervical cancer patients. TCGA data produced distinct clusters of both DNA repair genes and cervical cancer patients based upon the mRNA expression z-Scores. Higher than mean expression levels are depicted as red; below mean expression levels are depicted as blue. Annotation legend: (i) Gene cluster boundaries. (ii) Patient cluster boundaries. (iii) Somatic mutation levels (above mean mutation level: red; below mean: blue). (iv) HPV status (HPV16+: light blue; HPV18+: black). (v) Disease status (DF: light blue; RP: black; data missing: gray). (vi) Radiation therapy administered (yes: light blue; no: black; data missing: gray).

To explore the possibility of DNA repair differences between HPV16+ and HPV18+ patients, hierarchal clustering was performed on RNAseq data. The expression patterns of 546 DNA repair genes for 202 cervical cancer patients were included in the analysis, which yielded 12 gene clusters and seven patient clusters (Fig. 2). Upon comparing the clusters to each other, cluster F {CF} was the only cluster to have a significant difference in the percentage of patients who RP after receiving PRT (Supplementary Fig. 3A). 100% of the patients in CF who received radiation therapy were described as being disease free (compared to 27% rate of recurrence/progression for the patients in the other clusters). However, there was not a significant difference in the rate of HPV16 and HPV18 in CF (Supplementary Fig. 3B), which indicates that the difference in PRT outcome was not merely due to a bias in the number of HPV16+ patients. To further investigate this, the gene clusters with the highest and lowest mean expression levels were identified (Supplementary Fig. 3C). Intriguingly, cluster F2 {CF2} had the lowest mean expression (z-Score of −1.05) when compared to all the other patient/gene clusters and cluster F12 {CF12} had the highest mean expression (z-Score of 1.02). CF2 contained 47 genes and CF12 contained 69 genes whose expression (low/high respectively) could potentially confer increased susceptibility of cervical tumours to PRT (Supplementary Table 1).

**Figure 3:**
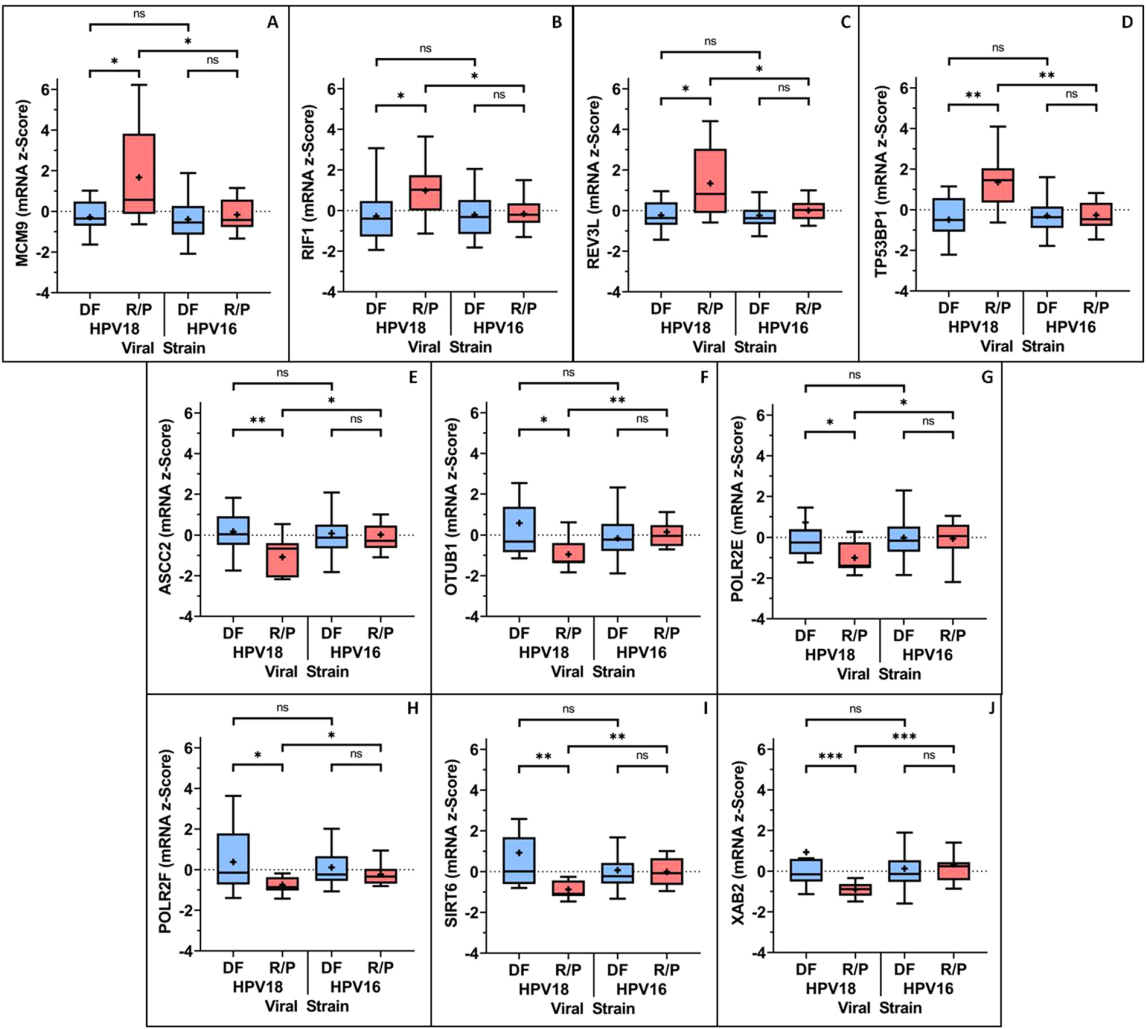
Expression levels of genes with significant differences between HPV18+ and HPV16+ cervical cancer patients. (**A**) MCM9, (**B**) RIF1, (**C**) REV3L and (**D**) TP53BP1 were more likely to be expressed at significantly (using the Mann-Whitney U test) higher levels in HPV18+ patients with cervical cancer who received PRT and later RP, than those who were DF and HPV16+ patients who later RP. TP53BP1 was the most significantly different of the four genes in this regard. Whereas, (**E**) ASCC2, (**F**) OTUB1, (**G**) POLR2E, (**H**) POLR2F, (**I**) SIRT6, (**J**) XAB2 were more likely to be expressed at significantly (using the Mann-Whitney U test) lower levels in HPV18+ patients with cervical cancer who received PRT and went on to RP, than the patients who were DF and also lower than HPV16+ patients who later RP.

Of the genes within CF2, four genes: MCM9, RIF1, REV3L and TP53BP1 displayed significantly higher expression in HPV18+ patients who RP following PRT, when compared to DF HPV18+ and HPV16+ patients (Fig. 3A–D). Whereas of the genes within CF12, six genes: ASCC2, OTUB1, POLR2E, POLR2F, SIRT6 and XAB2 displayed significantly lower expression in HPV18+ patients who experienced recurrence/progression following PRT when compared to DF HPV18+ and HPV16+ patients (Fig. 3E–J).

**Figure 4:**
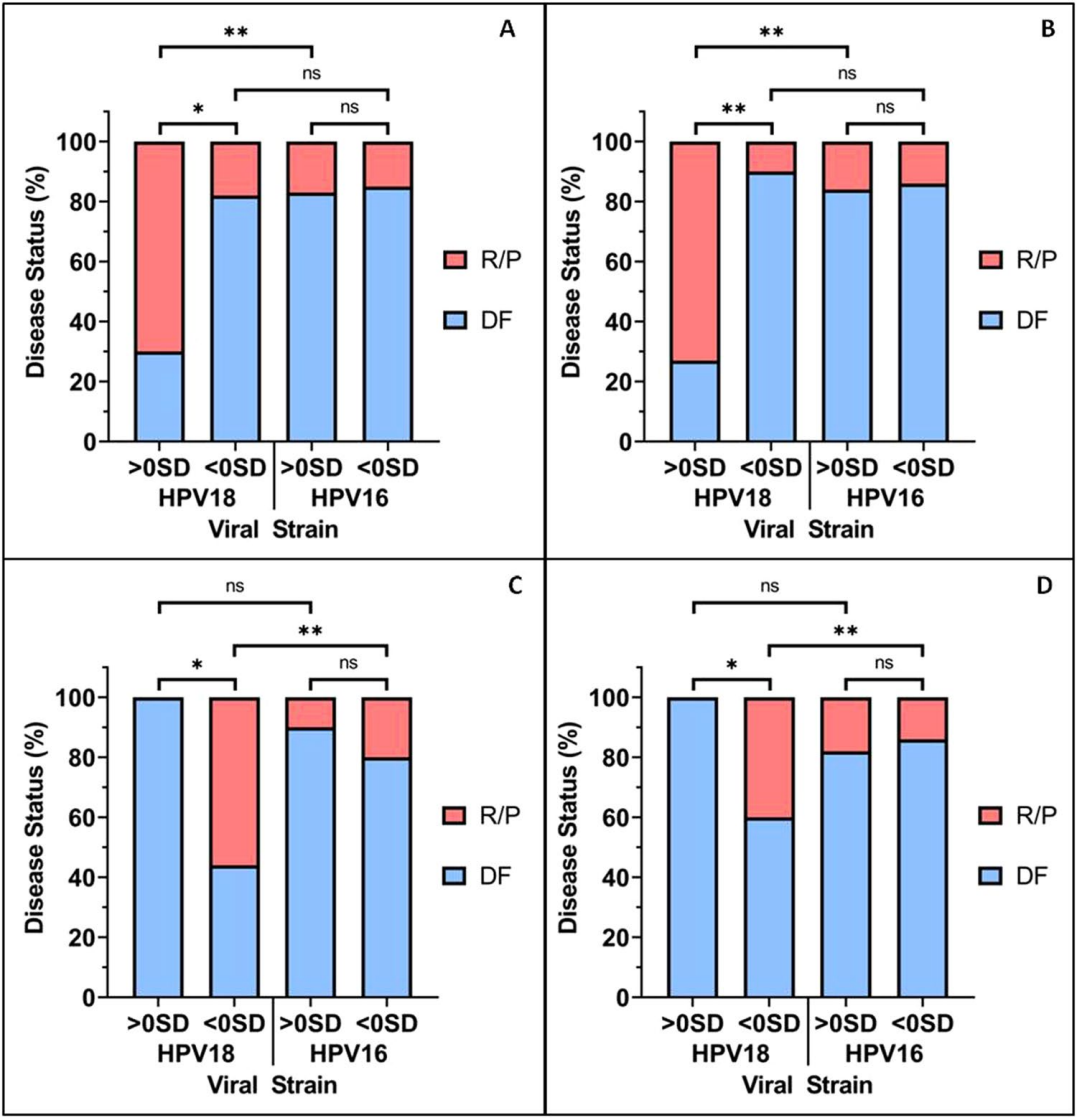
Expression levels of MCM9, TP53BP1, POLR2F and SIRT6 had a significant association with poorer prognosis after PRT for HPV18+ patients, but not HPV16+ patients. Of the four genes displayed in Fig. 3A–D, higher than mean {>0 SD} expression levels of (**A**) MCM9 and (**B**) TP53BP1 were associated with a significant increase in the percentage of HPV18+ patients experiencing recurrence/progression after PRT when compared to HPV18+ patients with below mean {<0 SD} expression levels. Whereas, of the six genes displayed in Fig. 3E–J, lower than mean expression levels of (**C**) POLR2F and (**D**) SIRT6 were associated with a significant increase in the percentage of HPV18+ patients who RP following PRT. For all four of the genes that were demonstrate to have difference in HPV18+ patients, there was no association with disease status for HPV16+ patients. A one-tailed Fisher’s exact test was used to generate the P values for this figure, because it was possible to predict the trend using data from Fig. 3.

Moreover, the cervical cancer patients were stratified based upon whether the four genes had z-Scores greater or lower than the mean expression level (i.e. a mean z-Score of zero). Patients with higher than mean expression levels of MCM9 (Fig. 4A) and TP53BP1 (Fig. 4B) were significantly more likely to experience recurrence/progression if HPV18+ when compared to HPV16+ patients. On the other hand, HPV18+ patients with below mean expression levels of POLR2F (Fig. 4C) and SIRT6 (Fig. 4D) were significantly more likely to experience disease recurrence/progression following PRT, when compared to HPV16+ patients. Of these genes, higher than mean expression of TP53BP1 was the most significantly linked to an increase of RP in HPV18+ patients, with an odds ratio of 24 (95% confidence interval of 1.8 to 288).

**Figure 5:**
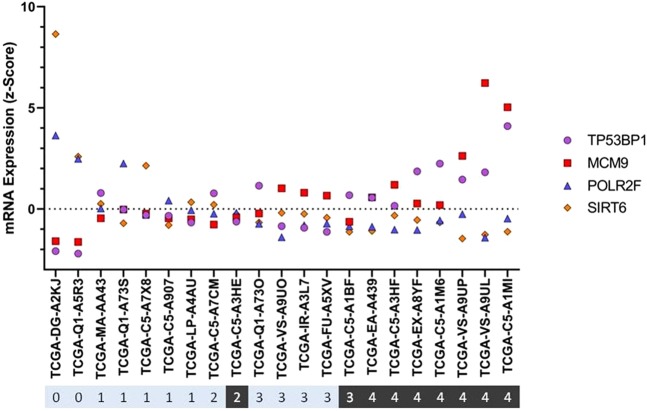
Predicative Value of HPV18+ Specific Genes. The HPV18+ cervical cancer patients who received PRT (out of a total of 38 HPV18+ patients, there were 21 patients who had disease status information available) were given a combined scored based upon the expression profile of TP53BP1 (z-Score of >0 = 1; <0 = 0), MCM9 (z-Score of >0 = 1; <0 = 0), POLR2F (z-Score of <0 = 1; >0 = 0) and SIRT6 (z-Score of <0 = 1; >0 = 0). For example, patients with above mean levels of TP53BP1 and MCM9, and below mean levels of POLR2F and SIRT6, would have the maximum score of four. The scores for each patient are displayed in a ribbon (zero to four), which also depicts their disease status (Black = RP, Light blue = DF).

These four genes could be used to form a scoring system (based upon the relationship of the genes to their respective means) that could provide a basis for a diagnostic test to predict whether a patient is likely to have a poor (RP) or good (DF) prognosis following PRT (Fig. 5). If a score of four were to be considered to be the threshold for prediction of a poor prognosis (zero to three, a predicted good prognosis; four, a predicted poor prognosis), the Fisher’s exact test would score this cohort with a P value of 0.0003. Moreover, a diagnostic test for poor prognosis based upon this scoring would have a sensitivity of 0.7778 (95% confidence interval [CI] 0.4526 to 0.9605), a specificity of 1.0000 (95% CI 0.7575 to 1.000), a positive predictive value of 1.0000 (95% CI 0.6457 to 1.000 and a negative predictive value of 0.8571 (95% CI of 0.6006 to 0.9746).

## Discussion

The cumulative results from this analysis of TCGA data demonstrate that there are indeed differences in the DNA repair processes within HPV16+ and HPV18+ cervical tumours and that these differences most likely affect the disease status of cervical cancer patients following PRT. In general, HPV16+ patients have a higher burden of somatic mutations when compared to HPV18+ patients. It is important to note that the mutations themselves were not analysed to determine their functional effect on disease outcome following PRT. However, the mutation level appears to be a symptom of the differences within the DNA repair processes, which ultimately cause the HPV16+ tumours to be more susceptible to PRT when compared to tumours in HPV18+ patients.

The RNAseq analysis of TCGA data enabled the identification of four genes that were significantly associated with a poorer (or less beneficial) outcome following PRT in HPV18+ cervical cancer patients when compared to HPV16+ patients. Expression levels of MCM9 and TP53BP1, when higher than the mean expression level, were significantly associated with a poorer outcome for cervical cancer patients if they were HPV18+, yet not HPV16+. Conversely, HPV18+ cervical cancer patients with expression of MCM9 and TP53BP1 at levels lower than the mean responded to radiation comparably to HPV16 patients (i.e. there was no significant difference). It is also noteworthy that REV3L was detected in the expression analysis, since its depletion has been associated with the suppression of the colony formation ability of multiple cervical cancer cell lines^21^, but it did not reach significance in the patient survival analysis. The second pair of genes, POLR2F and SIRT6, when expressed at expression levels lower than the mean were significantly associated with a poorer outcome for HPV18+ cervical cancer patients, which was not observed in HPV16+ patients.

The relationship between these four genes and the poorer prognosis for HPV18+ cervical cancer patients does require further exploration, including protein level validation in a larger independent cohort of HPV18+ patients. Especially when considering that all four of the genes have functions in addition to those related to DNA repair. But, nonetheless this study provides an explanation for the poorer prognosis associated with HPV18+ cervical cancer patients. It also raises the intriguing possibility of reducing the disparity between HPV16+ and HPV18+ patients following PRT, through the development of adjuvant PRT inhibitors for MCM9 and TP53BP1 (perhaps even some of the other CF2 genes). POLR2F and SIRT6 (or CF12 gene) activators could also be explored, though this would likely be more difficult than a MCM9 or TP53BP1 inhibitor. Such therapeutics could also prove useful for boosting the effectiveness of radiation therapy in other types/subtypes of cancers. Indeed, HPV18+ cervical cancer could prove a useful model for radiation resistance in other cancers.

Absent the development of targeted therapeutic interventions, these genes could prove useful as biomarkers for predicting which cervical cancer patients are more likely to have a poorer prognosis. The biomarkers in this instance could be used to guide the selection of patients by quantitative polymerase chain reaction for more rigorous follow-up/cancer screening and perhaps even alternative treatment regimens, such as those that incorporate postoperative chemotherapy. However, for these markers to be used in a clinical setting they would need to be validated in a larger cohort and the exact expression level for each gene would need to be defined (as opposed to the z-Scores presented herein). It is also possible that HPV18+ cervical cancer patients could benefit from the aforementioned additional clinical care based purely upon the presence of this viral strain alone, since they have a lower chance of overall survival compared to HPV16+ patients in general.

Moreover, a larger cohort would enable the assessment the impact of HPV integration patterns on cervical cancer patient prognosis following radiation therapy. This factor could be significant to gaining an understanding behind the HPV18 specific effect of the genes identified in this study. Differences between HPV16 and HPV18 integration that affect cervical cancer aggressiveness have previously been identified^22^. Specifically, that HPV18 tends to uniformly and completely integrated into the host cells, which subsequently often lack E2 gene expression^22–24^. On the other hand, HPV16 will integrate more variably with approximately 70% of cervical cancer cases having purely integrated DNA, 20% with both integrated and episomal DNA and 10% with only episomal DNA^23,24^. Integration patterns in within the TCGA HPV16+ tumours could account for some of the differences that cause increased susceptibility to PRT and possibly the disparate impact of the genes discussed in this study. Additionally, differences in the location of HPV16 and HPV18 into the host genome have been identified, for example HPV18 is more likely to integrate at the site of the *MYC* oncogene, which is located at 8q24.21^25^.

HPV18+ patients who have poor prognosis following PRT appear to have more robust DNA repair processes than those with a better prognosis. The functions of the genes most significantly linked to this feature could provide useful insights into this occurrence:

1. TP53BP1 plays an important role in the response to double strand DNA breaks and their repair through the promotion of non-homologous end joining (NHEJ) and is associated with response to radiation therapy^26,27^. The importance of TP53BP1 to this particular scenario is also supported by the significant expression patterns of RIF1 and OTUB1 in HPV18+ patients. RIF1 determines NHEJ pathway selection/activation in conjunction with TP53BP1^28,29^, whilst OTUB1 is capable of restricting TP53BP1 loading at sites of DNA damage^30,31^. Moreover, transient RIF1 silencing using short hairpin RNA has been shown to reduce the efficiency of HeLa cervical cancer cells (which are HPV18+) to form colonies and increased their sensitivity to Cisplatin^32^.
2. MCM9 is a component of the MCM8-MCM9 complex that facilitates double strand DNA repair through homologous recombination (HR)^33^.
3. POLR2F (in conjunction with POLR2E, whose expression was also significant) is involved in nucleotide excision repair^34^.
4. SIRT6 is generally associated with increased genomic stability and promotion of DNA end resection^35^, which would at first sight appear contradictory as it promotes more efficient DNA repair through HR. But, perhaps in this circumstance SIRT6 expression is indicative of single-strand annealing (rather than HR) promotion following DNA end resection, which is associated with mutagenic material deletions of DNA^36,37^.

Therefore, the data presented in this study suggests that HPV18+ cervical cancers could be specifically associated with a poorer prognosis after PRT due to a retained ability to activate the NHEJ and homologous recombination pathways. Whereas, it is possible that increased reliance upon nucleotide excision repair and single-strand annealing could be associated with a better prognosis through the conference of PRT susceptibility. The cluster analysis conducted herein indicates that there are similar expression features for both HPV16+ and HPV18+ patients who have a good prognosis following PRT. Yet, it is currently not clear why the expression differences observed in the four aforementioned genes are predominantly correlated with a higher chance of disease progression after PRT for HPV18+ cervical cancer patients and not HPV16+ patients.

Nevertheless, this study indicates that there could be some differences in these HPV strains with regard to the mechanisms through which cervical tumours develop, which in turn could cause a difference in DNA repair processes and PRT susceptibility. However, it is not readily apparent what the causative differences could be and further study in this area could be beneficial. Perhaps the most logical starting point would be the interactions between TP53BP1 and TP53, due to the known associations of the E6 viral oncogene and TP53^38^. This could be partially explored through immunoprecipitation of TP53BP1 to determine whether there is a difference in binding activity to TP53 between HPV16+ and HPV18+ cells. The resolution of this uncertainty could provide guidance upon whether the treatment of HPV16+ and HPV18+ cervical cancers should be tailored more specifically to the associated viral strain. It would also be interesting to determine whether this relationship also occurs in other types of cancers that are associated with HPV infection. Moreover, the resistance mechanisms of HPV18+ cancers relative to HPV16+ cancers could provide a useful model for the study of other types of cancer that are resistant to radiotherapy.

Additionally, the results from this study also support the possibility that DNA repair mechanisms and PRT response in cervical cancers that are associated with rarer strains of HPV could be distinctly affected. Strains other than HPV16 and HPV18 were not included in this study due to their relative rarity within the dataset and hence a lack of statistical power. Therefore, the development of cohorts focusing specifically on rarer strains of HPV could be useful to ensure all HPV patients are being treated with optimal treatment regimens. This could be especially important in the future if rarer strains not covered by the HPV vaccine begin to emerge as greater players in cervical cancer, since only nine of the 15 known oncogenic HPV strains are covered by the vaccine^5,6^.

## Conclusions

In summary, the analysis of TCGA data in this study suggests that HPV16+ patients could be more likely to have DNA repair defects than HPV18+ patients, due to the association with an increased number of somatic mutations. DNA repair defects could offer an explanation for the observation that PRT is more effective in general for HPV16+ cervical cancer patients, than HPV18+ patients. Moreover, increased expression levels of TP53BP1 and MCM9 and decreased expression levels of POLR2F and SIRT6 were found to be specifically associated with a poorer prognosis for HPV18+ (but not HPV16+) cervical cancer patients following PRT.

## Supplementary information

Supplementary Table 1.

Supplementary Information.

## Data Availability

All of the next generation sequencing datasets used in this study are publically available from the cBioportal and TCGA websites.
