# Supplementary material for: DNA repair gene expression is associated with differential prognosis between HPV16 and HPV18 positive cervical cancer patients following radiation therapy
Source: Sci Rep. 2020 Feb 17;10:2774. doi: 10.1038/s41598-020-59383-8 (PMC7026103; doi:10.1038/s41598-020-59383-8)
Supplement: Supplementary file 2 — Supplementary Information. [file 41598_2020_59383_MOESM2_ESM.docx]

**Title Page**

**Title:**

DNA repair gene expression is associated with differential prognosis between HPV16 and HPV18 positive cervical cancer patients following radiation therapy

**Author:**

Klarke M. Sample^a^

**Author Affiliation:**

1. The National Health Commission's Key Laboratory of Immunological Pulmonary Disease, Guizhou Provincial People’s Hospital, The Affiliated Hospital of Guizhou University.

**Corresponding Author Details:**

Dr. Klarke Michael Sample

Address: The National Health Commission's Key Laboratory of Immunological Pulmonary Disease, Guizhou Provincial People’s Hospital, Nanming District, Guiyang, Guizhou Province, China. 550002.

E-mail: [Klarke.Sample@outlook.com](mailto:Klarke.Sample@outlook.com)

**Supplementary Information:**

**
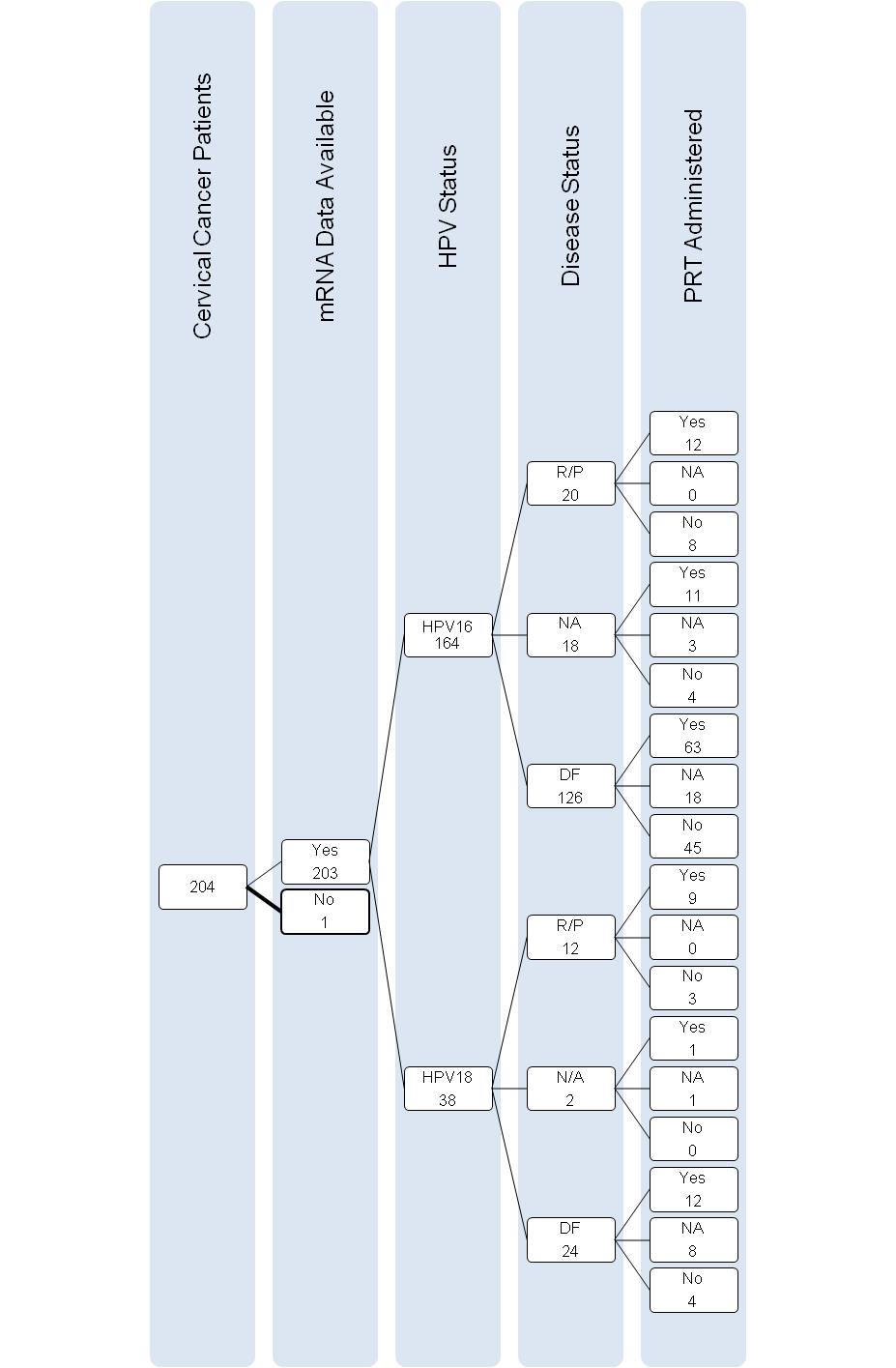
**

**Supplementary Figure 1: TCGA cohort characteristics for the Cervical Cancer Study.**

**
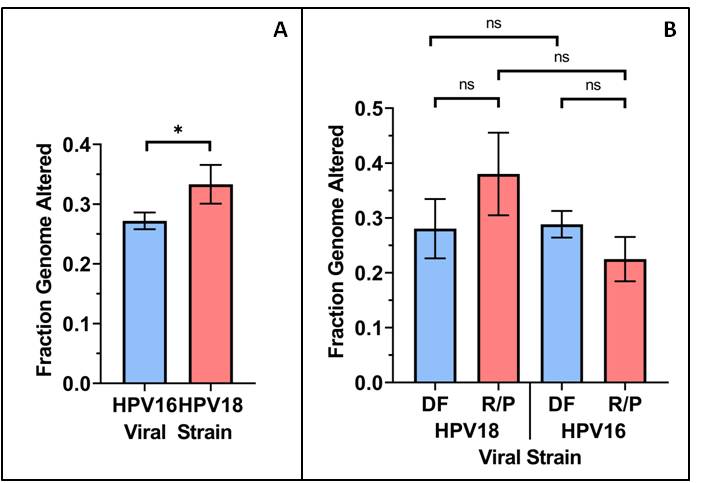
**

**Supplementary Figure 2: Fraction of genome altered in patients with HPV16+ and HPV18+ cervical cancers.**

A) A small significant difference in fraction of genome altered was observed between the HPV16+ and HPV18+ tumours in the TCGA cohort.

B) No significant association was observed between the fraction of genome altered and disease status in HPV16+ or HPV18+ cervical cancer patients who received PRT.

The statistical analysis in this figure was conducted using one-tailed Mann-Whitney U tests and the error bars were calculated using the standard error of the mean. The fraction of genome altered is defined as the log2 number of segments with a copy number alteration larger than 0.2 divided by the length of all segments measured.

**
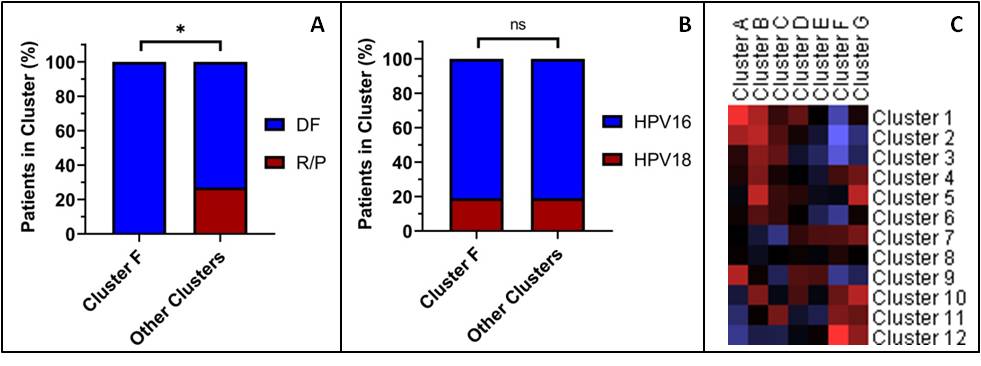
**

**Supplementary Figure 3: Patients in CF were more likely to be disease free than other patients.**

A) Patients in CF contained zero patients who experience RP after receiving PRT and were significantly (using Fisher’s exact test) more likely to be DF when compared to patients in the other clusters.

B) There was no significant difference (using Fisher’s exact test) in HPV status between the patients in CF and the other clusters.

C) Mean expression within the gene and patient clusters. The lowest overall mean expression score was -1.05 in cluster F2 (CF2); whilst the highest overall score was 1.02 in cluster F12 (CF12). Higher than mean expression levels are depicted as red; below mean expression levels are depicted as blue.

**Supplementary Table 1: RNAseq and Clinical Data for DNA repair genes in HPV16 and HPV18 patients from the Cervical Cancer Study (TCGA).**
